# *N*′-(2,4-Dichloro­benzyl­idene)-2-fluoro­benzohydrazide

**DOI:** 10.1107/S1600536810053249

**Published:** 2011-01-08

**Authors:** Hai-Tao Wang, Ling Yuan, Yi Nan, Rong Wang, Li Zhou, Yang Niu

**Affiliations:** a The Second Hospital of Jilin University, Changchun Jilin 130041, People’s Republic of China; b Pharmacy College of Ningxia Medical University, Yinchuan Ningxia 750004, People’s Republic of China; c Minority Traditional Medical Center of Minzu University of China, Beijing 100081, People’s Republic of China; d Traditional Chinese Medicine College of Ningxia Medical University, Yinchuan Ningxia 750004, People’s Republic of China

## Abstract

The mol­ecule of the title compound, C_14_H_9_Cl_2_FN_2_O, exists in a *trans* configuration with respect to the methyl­idene unit and the benzene rings form a dihedral angle of 8.1 (2)°. In the crystal, mol­ecules are linked through N—H⋯O hydrogen bonds into *C*(4) chains propagating in [100].

## Related literature

For related structures and background to the pharmacological properties of hydrazone compounds, see: Xu *et al.* (2011*a* ▶,*b* ▶). For reference bond-length values, see: Allen *et al.* (1987 ▶).
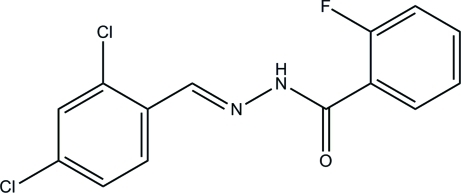


## Experimental

### 

#### Crystal data

C_14_H_9_Cl_2_FN_2_O
*M* *_r_* = 311.13
Monoclinic, 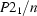

*a* = 7.2310 (14) Å
*b* = 26.145 (5) Å
*c* = 8.0590 (16) Å
β = 115.033 (3)°
*V* = 1380.5 (5) Å^3^
*Z* = 4
Mo *K*α radiation
μ = 0.48 mm^−1^
*T* = 298 K
0.23 × 0.21 × 0.20 mm

#### Data collection

Bruker SMART CCD diffractometer
Absorption correction: multi-scan (*SADABS*; Sheldrick, 1996 ▶) *T*_min_ = 0.898, *T*_max_ = 0.911
9842 measured reflections
2963 independent reflections
2236 reflections with *I* > 2σ(*I*)
*R*_int_ = 0.029

#### Refinement

*R*[*F* ^2^ > 2σ(*F* ^2^)] = 0.056
*wR*(*F* ^2^) = 0.163
*S* = 1.05
2963 reflections
184 parameters
1 restraint
H atoms treated by a mixture of independent and constrained refinement
Δρ_max_ = 0.66 e Å^−3^
Δρ_min_ = −0.27 e Å^−3^

### 

Data collection: *SMART* (Bruker, 1998 ▶); cell refinement: *SAINT* (Bruker, 1998 ▶); data reduction: *SAINT*; program(s) used to solve structure: *SHELXS97* (Sheldrick, 2008 ▶); program(s) used to refine structure: *SHELXL97* (Sheldrick, 2008 ▶); molecular graphics: *SHELXTL* (Sheldrick, 2008 ▶); software used to prepare material for publication: *SHELXTL*.

## Supplementary Material

Crystal structure: contains datablocks global, I. DOI: 10.1107/S1600536810053249/hb5775sup1.cif

Structure factors: contains datablocks I. DOI: 10.1107/S1600536810053249/hb5775Isup2.hkl

Additional supplementary materials:  crystallographic information; 3D view; checkCIF report

## Figures and Tables

**Table 1:** Hydrogen-bond geometry (Å, °)

*D*—H⋯*A*	*D*—H	H⋯*A*	*D*⋯*A*	*D*—H⋯*A*
N2—H2⋯O1^i^	0.89 (1)	2.19 (1)	3.054 (3)	162 (3)

Symmetry code: (i) 
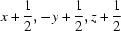
.

**Fig. 1.**
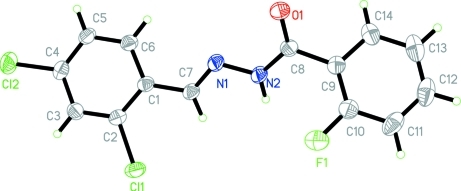
The molecular structure of the title compound, with displacement ellipsoids drawn at the 30% probability level.

**Fig. 2.**
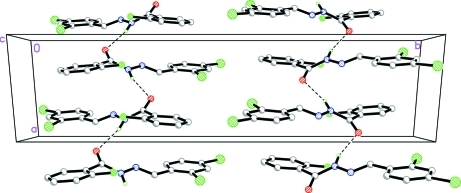
The one-dimensional chains structure along a axis. The intermolecular hydrogen bonds are shown as dashed lines.

## supplementary crystallographic information

### Comment

As a continuation of our recent work on the structures of hydrazone compounds (Xu *et al.*, 2011*a*,*b*), the title compound, (I), is reported.

The molecule of the compound, Fig. 1, exists in a *trans* configuration with respect to the methylidene unit. The molecule is twisted, with the dihedral angle between the two benzene rings of 8.1 (2)°. The torsion angle C7—N1—N2—C8 is 9.0 (3)°. The bond lengths are within normal ranges (Allen *et al.*, 1987).

In the crystal structure, molecules are linked through intermolecular N—H···O hydrogen bonds (Table 1), to form chains down the *a* axis (Fig. 2).

### Experimental

2,4-Dichlorobenzaldehyde (0.1 mmol, 17.4 mg) and 2-fluorobenzohydrazide (0.1 mmol, 15.4 mg) were mixed in ethanol (20 ml). The mixture was stirred at room temperature to give a clear colorless solution. Colorless well shaped blocks of the title compound were formed by gradual evaporation of the solvent over a period of five days at room temperature.

### Refinement

H2 atom was located from a difference Fourier map and refined isotropically, with N—H distance restrained to 0.90 (1) Å. The remaining H atoms were placed in geometrically idealized positions, with C—H = 0.93, and with U_iso_(H) = 1.2U_eq_(C).

### Crystal data

**Table:**

C_14_H_9_Cl_2_FN_2_O	*F*(000) = 632
*M**_r_* = 311.13	*D*_x_ = 1.497 Mg m^−^^3^
Monoclinic, *P*2_1_/*n*	Mo *K*α radiation, λ = 0.71073 Å
Hall symbol: -P 2yn	Cell parameters from 2459 reflections
*a* = 7.2310 (14) Å	θ = 2.7–25.0°
*b* = 26.145 (5) Å	µ = 0.48 mm^−^^1^
*c* = 8.0590 (16) Å	*T* = 298 K
β = 115.033 (3)°	Block, colorless
*V* = 1380.5 (5) Å^3^	0.23 × 0.21 × 0.20 mm
*Z* = 4	

### Data collection

**Table:**

Bruker SMART CCD diffractometer	2963 independent reflections
Radiation source: fine-focus sealed tube	2236 reflections with *I* > 2σ(*I*)
graphite	*R*_int_ = 0.029
ω scans	θ_max_ = 27.0°, θ_min_ = 1.6°
Absorption correction: multi-scan (*SADABS*; Sheldrick, 1996)	*h* = −9→9
*T*_min_ = 0.898, *T*_max_ = 0.911	*k* = −33→33
9842 measured reflections	*l* = −10→10

### Refinement

**Table:**

Refinement on *F*^2^	Primary atom site location: structure-invariant direct methods
Least-squares matrix: full	Secondary atom site location: difference Fourier map
*R*[*F*^2^ > 2σ(*F*^2^)] = 0.056	Hydrogen site location: inferred from neighbouring sites
*wR*(*F*^2^) = 0.163	H atoms treated by a mixture of independent and constrained refinement
*S* = 1.05	*w* = 1/[σ^2^(*F*_o_^2^) + (0.0831*P*)^2^ + 0.4914*P*] where *P* = (*F*_o_^2^ + 2*F*_c_^2^)/3
2963 reflections	(Δ/σ)_max_ < 0.001
184 parameters	Δρ_max_ = 0.66 e Å^−^^3^
1 restraint	Δρ_min_ = −0.27 e Å^−^^3^

### Special details

**Table:**

Geometry. All esds (except the esd in the dihedral angle between two l.s. planes) are estimated using the full covariance matrix. The cell esds are taken into account individually in the estimation of esds in distances, angles and torsion angles; correlations between esds in cell parameters are only used when they are defined by crystal symmetry. An approximate (isotropic) treatment of cell esds is used for estimating esds involving l.s. planes.
Refinement. Refinement of F^2^ against ALL reflections. The weighted R-factor wR and goodness of fit S are based on F^2^, conventional R-factors R are based on F, with F set to zero for negative F^2^. The threshold expression of F^2^ > 2sigma(F^2^) is used only for calculating R-factors(gt) etc. and is not relevant to the choice of reflections for refinement. R-factors based on F^2^ are statistically about twice as large as those based on F, and R- factors based on ALL data will be even larger.

### Fractional atomic coordinates and isotropic or equivalent isotropic displacement parameters (Å^2^)

**Table:**

	*x*	*y*	*z*	*U*_iso_*/*U*_eq_	
Cl1	0.86585 (15)	0.06215 (3)	0.64036 (10)	0.0753 (3)	
Cl2	0.71562 (15)	0.01533 (3)	−0.04771 (12)	0.0778 (3)	
F1	0.7577 (4)	0.25674 (8)	0.9556 (3)	0.0964 (7)	
N1	0.7523 (3)	0.21787 (8)	0.4805 (3)	0.0482 (5)	
N2	0.7956 (3)	0.24975 (8)	0.6285 (3)	0.0506 (6)	
O1	0.5927 (3)	0.31235 (8)	0.4527 (3)	0.0707 (6)	
C1	0.7912 (4)	0.13485 (10)	0.3799 (3)	0.0439 (6)	
C2	0.8097 (4)	0.08305 (11)	0.4195 (3)	0.0482 (6)	
C3	0.7842 (4)	0.04630 (10)	0.2903 (4)	0.0523 (6)	
H3	0.7953	0.0117	0.3203	0.063*	
C4	0.7417 (4)	0.06205 (11)	0.1141 (4)	0.0526 (7)	
C5	0.7245 (4)	0.11280 (11)	0.0675 (4)	0.0540 (7)	
H5	0.6977	0.1228	−0.0513	0.065*	
C6	0.7475 (4)	0.14872 (11)	0.1991 (4)	0.0517 (6)	
H6	0.7336	0.1832	0.1675	0.062*	
C7	0.8217 (4)	0.17291 (10)	0.5209 (3)	0.0479 (6)	
H7	0.8938	0.1640	0.6434	0.058*	
C8	0.7067 (4)	0.29579 (10)	0.6032 (3)	0.0479 (6)	
C9	0.7558 (4)	0.32662 (10)	0.7740 (3)	0.0475 (6)	
C10	0.7779 (4)	0.30700 (12)	0.9386 (4)	0.0581 (7)	
C11	0.8167 (5)	0.33677 (16)	1.0910 (4)	0.0761 (10)	
H11	0.8316	0.3221	1.2011	0.091*	
C12	0.8328 (5)	0.38854 (17)	1.0753 (5)	0.0832 (11)	
H12	0.8592	0.4095	1.1762	0.100*	
C13	0.8106 (5)	0.40961 (14)	0.9143 (6)	0.0796 (10)	
H13	0.8231	0.4448	0.9066	0.096*	
C14	0.7696 (4)	0.37967 (11)	0.7614 (5)	0.0615 (8)	
H14	0.7514	0.3947	0.6510	0.074*	
H2	0.886 (4)	0.2381 (12)	0.736 (2)	0.080*	

### Atomic displacement parameters (Å^2^)

**Table:**

	*U*^11^	*U*^22^	*U*^33^	*U*^12^	*U*^13^	*U*^23^
Cl1	0.1180 (7)	0.0635 (5)	0.0493 (4)	0.0186 (4)	0.0401 (5)	0.0144 (3)
Cl2	0.1043 (7)	0.0760 (6)	0.0646 (5)	−0.0129 (5)	0.0469 (5)	−0.0227 (4)
F1	0.143 (2)	0.0761 (14)	0.0794 (14)	0.0124 (13)	0.0558 (14)	0.0170 (11)
N1	0.0445 (12)	0.0506 (13)	0.0370 (11)	0.0004 (10)	0.0050 (9)	−0.0031 (9)
N2	0.0505 (13)	0.0511 (13)	0.0332 (11)	0.0077 (10)	0.0012 (9)	−0.0017 (9)
O1	0.0788 (14)	0.0604 (12)	0.0421 (11)	0.0151 (11)	−0.0044 (10)	0.0048 (9)
C1	0.0374 (12)	0.0507 (14)	0.0375 (13)	0.0004 (11)	0.0099 (10)	0.0028 (11)
C2	0.0493 (14)	0.0553 (15)	0.0397 (13)	0.0055 (12)	0.0186 (11)	0.0053 (11)
C3	0.0584 (16)	0.0474 (15)	0.0552 (16)	−0.0029 (12)	0.0281 (14)	−0.0016 (12)
C4	0.0500 (15)	0.0631 (17)	0.0470 (15)	−0.0085 (12)	0.0227 (12)	−0.0117 (12)
C5	0.0581 (16)	0.0636 (17)	0.0360 (13)	−0.0107 (13)	0.0158 (12)	−0.0011 (12)
C6	0.0510 (15)	0.0538 (15)	0.0430 (14)	−0.0041 (12)	0.0127 (12)	0.0047 (12)
C7	0.0445 (14)	0.0536 (15)	0.0369 (13)	0.0043 (12)	0.0087 (11)	0.0011 (11)
C8	0.0409 (13)	0.0505 (15)	0.0410 (14)	0.0012 (11)	0.0064 (11)	0.0019 (11)
C9	0.0321 (12)	0.0548 (15)	0.0437 (14)	0.0054 (11)	0.0044 (10)	−0.0010 (11)
C10	0.0510 (16)	0.0625 (18)	0.0527 (16)	0.0142 (13)	0.0140 (13)	0.0033 (14)
C11	0.063 (2)	0.109 (3)	0.0447 (17)	0.0231 (19)	0.0124 (14)	−0.0059 (17)
C12	0.062 (2)	0.096 (3)	0.072 (2)	0.0140 (19)	0.0088 (18)	−0.035 (2)
C13	0.061 (2)	0.064 (2)	0.099 (3)	0.0019 (16)	0.0197 (19)	−0.025 (2)
C14	0.0491 (16)	0.0514 (16)	0.073 (2)	0.0019 (12)	0.0147 (14)	−0.0084 (14)

### Geometric parameters (Å, °)

**Table:**

Cl1—C2	1.738 (3)	C5—C6	1.373 (4)
Cl2—C4	1.738 (3)	C5—H5	0.9300
F1—C10	1.336 (4)	C6—H6	0.9300
N1—C7	1.265 (3)	C7—H7	0.9300
N1—N2	1.379 (3)	C8—C9	1.503 (4)
N2—C8	1.339 (3)	C9—C10	1.367 (4)
N2—H2	0.893 (10)	C9—C14	1.397 (4)
O1—C8	1.222 (3)	C10—C11	1.379 (4)
C1—C2	1.385 (4)	C11—C12	1.369 (6)
C1—C6	1.401 (4)	C11—H11	0.9300
C1—C7	1.455 (3)	C12—C13	1.355 (6)
C2—C3	1.371 (4)	C12—H12	0.9300
C3—C4	1.382 (4)	C13—C14	1.382 (5)
C3—H3	0.9300	C13—H13	0.9300
C4—C5	1.370 (4)	C14—H14	0.9300
			
C7—N1—N2	114.8 (2)	N1—C7—H7	119.3
C8—N2—N1	119.5 (2)	C1—C7—H7	119.3
C8—N2—H2	124 (2)	O1—C8—N2	123.3 (2)
N1—N2—H2	116 (2)	O1—C8—C9	121.2 (2)
C2—C1—C6	116.8 (2)	N2—C8—C9	115.5 (2)
C2—C1—C7	121.4 (2)	C10—C9—C14	117.4 (3)
C6—C1—C7	121.9 (2)	C10—C9—C8	125.0 (3)
C3—C2—C1	122.8 (2)	C14—C9—C8	117.5 (2)
C3—C2—Cl1	117.1 (2)	F1—C10—C9	119.7 (3)
C1—C2—Cl1	120.1 (2)	F1—C10—C11	117.0 (3)
C2—C3—C4	118.1 (3)	C9—C10—C11	123.3 (3)
C2—C3—H3	120.9	C12—C11—C10	118.0 (3)
C4—C3—H3	120.9	C12—C11—H11	121.0
C5—C4—C3	121.7 (2)	C10—C11—H11	121.0
C5—C4—Cl2	120.4 (2)	C13—C12—C11	120.7 (3)
C3—C4—Cl2	117.9 (2)	C13—C12—H12	119.7
C4—C5—C6	118.9 (2)	C11—C12—H12	119.7
C4—C5—H5	120.5	C12—C13—C14	121.1 (3)
C6—C5—H5	120.5	C12—C13—H13	119.4
C5—C6—C1	121.8 (3)	C14—C13—H13	119.4
C5—C6—H6	119.1	C13—C14—C9	119.5 (3)
C1—C6—H6	119.1	C13—C14—H14	120.3
N1—C7—C1	121.4 (2)	C9—C14—H14	120.3

### Hydrogen-bond geometry (Å, °)

**Table:**

*D*—H···*A*	*D*—H	H···*A*	*D*···*A*	*D*—H···*A*
N2—H2···O1^i^	0.89 (1)	2.19 (1)	3.054 (3)	162 (3)

Symmetry codes: (i) *x*+1/2, −*y*+1/2, *z*+1/2.
